# 5′′-(4-Meth­oxy­benzyl­idene)-7′-(4-meth­oxy­phen­yl)-1′′-methyl-5′,6′,7′,7a’-tetra­hydro­dispiro­[acenaphthene-1,5′-pyrrolo­[1,2-*c*][1,3]thia­zole-6′,3′′-piperidine]-2,4′′-dione

**DOI:** 10.1107/S1600536811045934

**Published:** 2011-11-05

**Authors:** J. Suresh, R. Vishnupriya, R. Ranjith Kumar, S. Sivakumar, P. L. Nilantha Lakshman

**Affiliations:** a Department of Physics, The Madura College, Madurai 625 011, India; b Department of Organic Chemistry, School of Chemistry, Madurai Kamaraj University, Madurai 625 021, India; c Department of Food Science and Technology, University of Ruhuna, Mapalana, Kamburupitiya 81100, Sri Lanka

## Abstract

In the title compound, C_37_H_34_N_2_O_4_S, the piperidine ring adopts a half-chair conformation. The thia­zole ring adopts a slightly twisted envelope conformation and the pyrrole ring adopts an envelope conformation; in each case, the C atom linking the rings is the flap atom. An intra­molecular C—H⋯O inter­action is noted. The crystal structure is stabilized by C—H⋯O and C—H⋯π inter­actions.

## Related literature

For background to the importance of spiro compounds, see: Kobayashi *et al.* (1991 ▶); James *et al.* (1991 ▶); Caramella & Grunanger (1984 ▶). For hydrogen-bond motifs, see: Bernstein *et al.*, 1995 ▶,
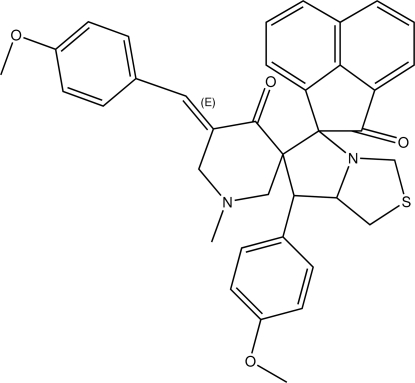


## Experimental

### 

#### Crystal data

C_37_H_34_N_2_O_4_S
*M* *_r_* = 602.72
Monoclinic, 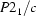

*a* = 14.6229 (6) Å
*b* = 15.8759 (6) Å
*c* = 15.0284 (5) Å
β = 115.907 (2)°
*V* = 3138.3 (2) Å^3^
*Z* = 4
Mo *K*α radiation
μ = 0.15 mm^−1^
*T* = 293 K
0.23 × 0.21 × 0.18 mm

#### Data collection

Bruker Kappa APEXII diffractometer
Absorption correction: multi-scan (*SADABS*; Sheldrick, 1996 ▶) *T*_min_ = 0.967, *T*_max_ = 0.974
44023 measured reflections
10327 independent reflections
6691 reflections with *I* > 2σ(*I*)
*R*_int_ = 0.031

#### Refinement

*R*[*F* ^2^ > 2σ(*F* ^2^)] = 0.051
*wR*(*F* ^2^) = 0.154
*S* = 1.01
10327 reflections
400 parameters
H-atom parameters constrained
Δρ_max_ = 0.43 e Å^−3^
Δρ_min_ = −0.33 e Å^−3^

### 

Data collection: *APEX2* (Bruker, 2004 ▶); cell refinement: *SAINT* (Bruker, 2004 ▶); data reduction: *SAINT*; program(s) used to solve structure: *SHELXS97* (Sheldrick, 2008 ▶); program(s) used to refine structure: *SHELXL97* (Sheldrick, 2008 ▶); molecular graphics: *PLATON* (Spek, 2009 ▶); software used to prepare material for publication: *SHELXL97*.

## Supplementary Material

Crystal structure: contains datablock(s) global, I. DOI: 10.1107/S1600536811045934/tk5009sup1.cif

Structure factors: contains datablock(s) I. DOI: 10.1107/S1600536811045934/tk5009Isup2.hkl

Additional supplementary materials:  crystallographic information; 3D view; checkCIF report

## Figures and Tables

**Table 1:** Hydrogen-bond geometry (Å, °) *Cg*1 and *Cg*2 are the centroids of the C17–C22 and C71–C76 rings, respectively.

*D*—H⋯*A*	*D*—H	H⋯*A*	*D*⋯*A*	*D*—H⋯*A*
C76—H76⋯O2^i^	0.93	2.38	3.263 (2)	159
C34—H34⋯O1^ii^	0.93	2.53	3.453 (2)	171
C10—H10*B*⋯O2	0.97	2.53	3.173 (2)	124
C2—H2*A*⋯*Cg*1^iii^	0.97	2.73	3.658 (2)	160
C38—H38*B*⋯*Cg*2^iv^	0.96	2.93	3.730 (2)	141

Symmetry codes: (i) 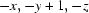; (ii) 
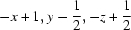
; (iii) 
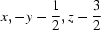
; (iv) 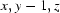.

**Fig. 1.**
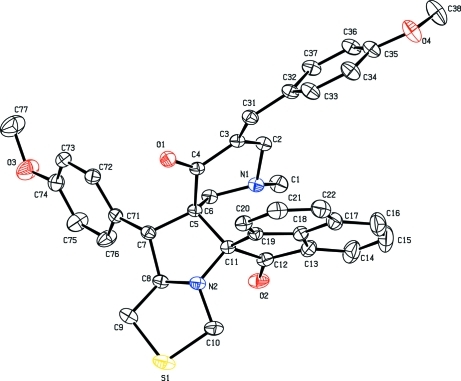
The molecular structure of (I), showing 30% probability displacement ellipsoids and the atom-numbering scheme. Hydrogen atoms have been omitted for clarity.

**Fig. 2.**
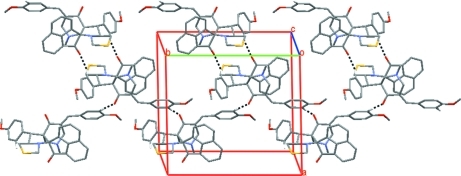
A partial packing diagram for (I). The C—H···O,π interactions are shown as dashed lines.

## supplementary crystallographic information

### Comment

Spiro-compounds represent an important class of naturally occurring substances, which in many cases exhibit important biological properties (Kobayashi *et al.*, 1991; James *et al.*, 1991). 1,3-Dipolar cycloaddition reactions are widely used for the construction of spiro-compounds (Caramella & Grunanger, 1984).

In the title compound (Fig. 1), the six-membered piperidine ring adopts a half-chair conformation with atoms N1 and C5 deviating by -0.532 (2) and -0.588 (2) Å, respectively, from the least-squares plane defined by atoms C2/C3/C4/C6. In the pyrrole thiazole fused ring system, the pyrrole ring has an envelope conformation, and the thiazole ring is in a twisted envelope conformation with C8 atoms being the flap atom in both of these envelopes. The twisted envelope conformation of the thiazole ring may be due to the intramolecular C10—H10B···O2 interaction (Table 1). Similarly the orientation of the 4-methoxyphenyl substituent with respect to the attached piperidine ring may be influenced by the intermolecular C34—H34···O1 interaction (Table 1). The dihedral angle between the methoxyphenyl rings are 86.4 (1)°, and these rings form dihedral angles of 59.1 (1) and 39.8 (1)° with the acenaphthene group.

The C72—H72···O2 hydrogen bond connect two centrosymmetrically related molecules and generate the graph set motif *R*_2_^2^(16) (Bernstein *et al.*, 1995), Fig. 2. These dimers are connected into a zigzag chain by C34—H34···O1 hydrogen bonds (Fig. 2). In addition, there are two weak C—H···π interactions, Table 1.

### Experimental

1-Methyl-3,5-bis[(*E*)-4-methoxyphenylmethylidene]tetrahydro-4(1*H*)-pyridinone (1 mmol), acenaphthenequinone (0.182 g, 1 mmol) and 1,3-thiazolane-4-carboxylic acid (0.133 g, 1 mmol) were dissolved in methanol (10 ml) and refluxed for 30 min. After completion of the reaction, as evident from TLC, the mixture was poured into water (50 ml). The precipitated solid was filtered and washed with water (100 ml) to obtain the pure product as a solid. The product was recrystallized from ethyl acetate to obtain suitable crystals for the X-ray analysis. Melting point: 479 K; Yield: 89%.

### Refinement

H atoms were placed at calculated positions and allowed to ride on their carrier atoms with C—H = 0.93–0.98 Å. *U*_iso_ = 1.2*U*_eq_(C) for CH_2_ and CH groups, and *U*_iso_ = 1.5*U*_eq_(C) for CH_3_ groups.

### Crystal data

**Table:**

C_37_H_34_N_2_O_4_S	*F*(000) = 1272
*M**_r_* = 602.72	*D*_x_ = 1.276 Mg m^−^^3^
Monoclinic, *P*2_1_/*c*	Mo *K*α radiation, λ = 0.71073 Å
Hall symbol: -P 2ybc	Cell parameters from 2000 reflections
*a* = 14.6229 (6) Å	θ = 2–31°
*b* = 15.8759 (6) Å	µ = 0.15 mm^−^^1^
*c* = 15.0284 (5) Å	*T* = 293 K
β = 115.907 (2)°	Block, colourless
*V* = 3138.3 (2) Å^3^	0.23 × 0.21 × 0.18 mm
*Z* = 4	

### Data collection

**Table:**

Bruker Kappa APEXII diffractometer	10327 independent reflections
Radiation source: fine-focus sealed tube	6691 reflections with *I* > 2σ(*I*)
graphite	*R*_int_ = 0.031
Detector resolution: 0 pixels mm^-1^	θ_max_ = 31.4°, θ_min_ = 1.6°
ω and φ scans	*h* = −21→21
Absorption correction: multi-scan (*SADABS*; Sheldrick, 1996)	*k* = −23→23
*T*_min_ = 0.967, *T*_max_ = 0.974	*l* = −21→14
44023 measured reflections	

### Refinement

**Table:**

Refinement on *F*^2^	Secondary atom site location: difference Fourier map
Least-squares matrix: full	Hydrogen site location: inferred from neighbouring sites
*R*[*F*^2^ > 2σ(*F*^2^)] = 0.051	H-atom parameters constrained
*wR*(*F*^2^) = 0.154	*w* = 1/[σ^2^(*F*_o_^2^) + (0.0699*P*)^2^ + 0.9424*P*] where *P* = (*F*_o_^2^ + 2*F*_c_^2^)/3
*S* = 1.01	(Δ/σ)_max_ < 0.001
10327 reflections	Δρ_max_ = 0.43 e Å^−^^3^
400 parameters	Δρ_min_ = −0.33 e Å^−^^3^
0 restraints	Extinction correction: *SHELXL97* (Sheldrick, 2008), Fc^*^=kFc[1+0.001xFc^2^λ^3^/sin(2θ)]^-1/4^
Primary atom site location: structure-invariant direct methods	Extinction coefficient: 0.0015 (5)

### Special details

**Table:**

Geometry. All e.s.d.'s (except the e.s.d. in the dihedral angle between two l.s. planes) are estimated using the full covariance matrix. The cell e.s.d.'s are taken into account individually in the estimation of e.s.d.'s in distances, angles and torsion angles; correlations between e.s.d.'s in cell parameters are only used when they are defined by crystal symmetry. An approximate (isotropic) treatment of cell e.s.d.'s is used for estimating e.s.d.'s involving l.s. planes.
Refinement. Refinement of *F*^2^ against ALL reflections. The weighted *R*-factor *wR* and goodness of fit *S* are based on *F*^2^, conventional *R*-factors *R* are based on *F*, with *F* set to zero for negative *F*^2^. The threshold expression of *F*^2^ > σ(*F*^2^) is used only for calculating *R*-factors(gt) *etc*. and is not relevant to the choice of reflections for refinement. *R*-factors based on *F*^2^ are statistically about twice as large as those based on *F*, and *R*- factors based on ALL data will be even larger.

### Fractional atomic coordinates and isotropic or equivalent isotropic displacement parameters (Å^2^)

**Table:**

	*x*	*y*	*z*	*U*_iso_*/*U*_eq_	
C1	0.05702 (12)	0.26172 (11)	−0.14785 (12)	0.0458 (4)	
H1A	0.0460	0.2022	−0.1463	0.069*	
H1B	0.0656	0.2753	−0.2060	0.069*	
H1C	−0.0005	0.2917	−0.1490	0.069*	
C2	0.23369 (10)	0.23225 (9)	−0.04797 (11)	0.0349 (3)	
H2A	0.2414	0.2326	−0.1089	0.042*	
H2B	0.2190	0.1749	−0.0361	0.042*	
C3	0.33233 (10)	0.25945 (9)	0.03580 (10)	0.0320 (3)	
C4	0.34405 (10)	0.34748 (9)	0.07216 (10)	0.0302 (3)	
C5	0.24559 (10)	0.39442 (8)	0.05210 (10)	0.0293 (3)	
C6	0.17140 (10)	0.37496 (9)	−0.05547 (10)	0.0330 (3)	
H6A	0.1100	0.4082	−0.0752	0.040*	
H6B	0.2023	0.3882	−0.0993	0.040*	
C7	0.26211 (10)	0.49022 (9)	0.07440 (10)	0.0320 (3)	
H7	0.3297	0.4974	0.1296	0.038*	
C8	0.18394 (11)	0.50890 (9)	0.11325 (11)	0.0338 (3)	
H8	0.1151	0.5082	0.0590	0.041*	
C9	0.19802 (13)	0.58458 (10)	0.17907 (13)	0.0452 (4)	
H9A	0.1686	0.6346	0.1400	0.054*	
H9B	0.2695	0.5947	0.2211	0.054*	
C10	0.13378 (13)	0.44349 (11)	0.22827 (13)	0.0448 (4)	
H10A	0.1615	0.4117	0.2895	0.054*	
H10B	0.0660	0.4228	0.1864	0.054*	
C11	0.19993 (10)	0.36021 (9)	0.12522 (10)	0.0308 (3)	
C12	0.09191 (11)	0.31906 (10)	0.06612 (11)	0.0379 (3)	
C13	0.09879 (12)	0.22999 (11)	0.09739 (13)	0.0444 (4)	
C14	0.02966 (17)	0.16525 (14)	0.06977 (17)	0.0671 (6)	
H14	−0.0365	0.1729	0.0214	0.081*	
C15	0.0619 (2)	0.08708 (16)	0.1169 (2)	0.0831 (8)	
H15	0.0160	0.0425	0.0980	0.100*	
C16	0.1577 (2)	0.07374 (14)	0.18930 (19)	0.0738 (7)	
H16	0.1755	0.0208	0.2182	0.089*	
C17	0.23036 (16)	0.13913 (11)	0.22093 (14)	0.0518 (4)	
C18	0.19704 (12)	0.21657 (10)	0.17185 (12)	0.0390 (3)	
C19	0.25791 (11)	0.28929 (9)	0.19501 (10)	0.0327 (3)	
C20	0.35303 (11)	0.28621 (10)	0.27196 (11)	0.0394 (3)	
H20	0.3939	0.3340	0.2911	0.047*	
C21	0.38795 (14)	0.20853 (12)	0.32189 (13)	0.0512 (4)	
H21	0.4530	0.2060	0.3736	0.061*	
C22	0.33004 (16)	0.13745 (12)	0.29711 (14)	0.0570 (5)	
H22	0.3566	0.0875	0.3308	0.068*	
C31	0.40937 (11)	0.20670 (9)	0.08693 (11)	0.0368 (3)	
H31	0.4669	0.2315	0.1360	0.044*	
C32	0.41493 (11)	0.11611 (9)	0.07593 (11)	0.0375 (3)	
C33	0.46607 (12)	0.06725 (10)	0.16063 (12)	0.0444 (4)	
H33	0.5013	0.0939	0.2213	0.053*	
C34	0.46569 (14)	−0.01911 (11)	0.15661 (13)	0.0490 (4)	
H34	0.4987	−0.0503	0.2143	0.059*	
C35	0.41601 (13)	−0.05974 (10)	0.06629 (13)	0.0450 (4)	
C36	0.36961 (13)	−0.01298 (11)	−0.01935 (12)	0.0454 (4)	
H36	0.3388	−0.0398	−0.0804	0.055*	
C37	0.36913 (12)	0.07357 (10)	−0.01411 (12)	0.0421 (3)	
H37	0.3374	0.1045	−0.0722	0.050*	
C38	0.3592 (2)	−0.18946 (14)	−0.01891 (18)	0.0799 (7)	
H38A	0.2898	−0.1708	−0.0455	0.120*	
H38B	0.3622	−0.2488	−0.0056	0.120*	
H38C	0.3859	−0.1786	−0.0659	0.120*	
C71	0.25792 (11)	0.54436 (9)	−0.01021 (11)	0.0345 (3)	
C72	0.34035 (12)	0.54987 (10)	−0.03242 (13)	0.0413 (3)	
H72	0.3997	0.5210	0.0070	0.050*	
C73	0.33715 (13)	0.59696 (10)	−0.11150 (14)	0.0459 (4)	
H73	0.3933	0.5987	−0.1252	0.055*	
C74	0.25059 (14)	0.64111 (11)	−0.16955 (13)	0.0475 (4)	
C75	0.16901 (14)	0.63866 (13)	−0.14731 (15)	0.0588 (5)	
H75	0.1108	0.6694	−0.1854	0.071*	
C76	0.17252 (13)	0.59095 (12)	−0.06887 (14)	0.0511 (4)	
H76	0.1163	0.5901	−0.0552	0.061*	
C77	0.3074 (2)	0.68197 (18)	−0.28915 (19)	0.0842 (8)	
H77A	0.3743	0.6966	−0.2403	0.126*	
H77B	0.2883	0.7186	−0.3453	0.126*	
H77C	0.3072	0.6247	−0.3096	0.126*	
N1	0.14777 (8)	0.28580 (8)	−0.06042 (9)	0.0336 (3)	
N2	0.19908 (9)	0.43700 (7)	0.17839 (9)	0.0341 (3)	
O1	0.42678 (7)	0.38018 (7)	0.11706 (8)	0.0411 (3)	
O2	0.01535 (8)	0.35883 (8)	0.01723 (9)	0.0493 (3)	
O3	0.23835 (13)	0.69080 (10)	−0.24882 (12)	0.0747 (5)	
O4	0.41766 (11)	−0.14553 (8)	0.06995 (10)	0.0630 (4)	
S1	0.13145 (4)	0.55591 (3)	0.25237 (3)	0.05553 (14)	

### Atomic displacement parameters (Å^2^)

**Table:**

	*U*^11^	*U*^22^	*U*^33^	*U*^12^	*U*^13^	*U*^23^
C1	0.0365 (7)	0.0518 (9)	0.0387 (8)	0.0007 (7)	0.0068 (6)	−0.0070 (7)
C2	0.0350 (7)	0.0359 (7)	0.0325 (7)	0.0051 (5)	0.0136 (6)	−0.0014 (6)
C3	0.0313 (6)	0.0342 (7)	0.0328 (7)	0.0042 (5)	0.0162 (5)	0.0001 (5)
C4	0.0293 (6)	0.0335 (7)	0.0290 (6)	0.0048 (5)	0.0140 (5)	0.0025 (5)
C5	0.0284 (6)	0.0310 (6)	0.0291 (6)	0.0042 (5)	0.0130 (5)	0.0008 (5)
C6	0.0330 (6)	0.0357 (7)	0.0290 (6)	0.0054 (5)	0.0123 (5)	0.0014 (5)
C7	0.0293 (6)	0.0318 (6)	0.0334 (7)	0.0043 (5)	0.0124 (5)	0.0002 (5)
C8	0.0337 (6)	0.0356 (7)	0.0319 (7)	0.0069 (5)	0.0142 (6)	0.0002 (5)
C9	0.0503 (9)	0.0400 (8)	0.0439 (9)	0.0091 (7)	0.0192 (7)	−0.0052 (7)
C10	0.0494 (9)	0.0528 (10)	0.0387 (8)	0.0081 (7)	0.0253 (7)	−0.0010 (7)
C11	0.0288 (6)	0.0349 (7)	0.0290 (6)	0.0028 (5)	0.0130 (5)	0.0001 (5)
C12	0.0309 (6)	0.0492 (9)	0.0357 (7)	−0.0009 (6)	0.0166 (6)	−0.0049 (6)
C13	0.0453 (8)	0.0495 (9)	0.0435 (9)	−0.0110 (7)	0.0242 (7)	−0.0048 (7)
C14	0.0651 (12)	0.0733 (14)	0.0624 (13)	−0.0302 (11)	0.0275 (10)	−0.0103 (10)
C15	0.112 (2)	0.0616 (14)	0.0806 (17)	−0.0428 (14)	0.0462 (16)	−0.0091 (12)
C16	0.113 (2)	0.0443 (11)	0.0741 (15)	−0.0153 (11)	0.0498 (15)	0.0022 (10)
C17	0.0792 (13)	0.0378 (8)	0.0513 (10)	0.0007 (8)	0.0404 (10)	0.0045 (7)
C18	0.0485 (8)	0.0378 (8)	0.0381 (8)	−0.0016 (6)	0.0258 (7)	0.0001 (6)
C19	0.0369 (7)	0.0349 (7)	0.0308 (7)	0.0048 (5)	0.0191 (6)	0.0023 (5)
C20	0.0397 (7)	0.0450 (8)	0.0341 (7)	0.0061 (6)	0.0168 (6)	0.0059 (6)
C21	0.0552 (10)	0.0584 (11)	0.0413 (9)	0.0192 (8)	0.0223 (8)	0.0169 (8)
C22	0.0809 (13)	0.0465 (10)	0.0533 (11)	0.0190 (9)	0.0383 (10)	0.0184 (8)
C31	0.0330 (7)	0.0368 (7)	0.0387 (8)	0.0053 (5)	0.0138 (6)	−0.0009 (6)
C32	0.0354 (7)	0.0369 (7)	0.0376 (8)	0.0097 (6)	0.0135 (6)	−0.0002 (6)
C33	0.0446 (8)	0.0419 (8)	0.0353 (8)	0.0121 (6)	0.0068 (6)	−0.0036 (6)
C34	0.0579 (10)	0.0411 (9)	0.0379 (8)	0.0143 (7)	0.0116 (7)	0.0025 (7)
C35	0.0469 (8)	0.0376 (8)	0.0467 (9)	0.0087 (6)	0.0168 (7)	−0.0031 (7)
C36	0.0472 (8)	0.0455 (9)	0.0371 (8)	0.0092 (7)	0.0124 (7)	−0.0078 (7)
C37	0.0442 (8)	0.0456 (8)	0.0332 (7)	0.0126 (6)	0.0140 (6)	0.0010 (6)
C38	0.0941 (17)	0.0459 (11)	0.0766 (15)	−0.0010 (11)	0.0160 (13)	−0.0201 (10)
C71	0.0343 (7)	0.0306 (7)	0.0400 (8)	0.0024 (5)	0.0175 (6)	0.0016 (6)
C72	0.0360 (7)	0.0371 (8)	0.0536 (9)	0.0044 (6)	0.0221 (7)	0.0036 (7)
C73	0.0481 (9)	0.0406 (8)	0.0614 (10)	−0.0004 (7)	0.0354 (8)	0.0018 (7)
C74	0.0567 (10)	0.0412 (8)	0.0510 (10)	0.0024 (7)	0.0294 (8)	0.0080 (7)
C75	0.0494 (10)	0.0685 (12)	0.0613 (12)	0.0204 (9)	0.0268 (9)	0.0293 (10)
C76	0.0396 (8)	0.0628 (11)	0.0564 (10)	0.0148 (7)	0.0262 (8)	0.0214 (9)
C77	0.0949 (18)	0.102 (2)	0.0765 (16)	0.0126 (15)	0.0570 (15)	0.0296 (14)
N1	0.0288 (5)	0.0379 (6)	0.0302 (6)	0.0027 (4)	0.0094 (4)	−0.0024 (5)
N2	0.0377 (6)	0.0358 (6)	0.0315 (6)	0.0070 (5)	0.0177 (5)	0.0005 (5)
O1	0.0289 (5)	0.0402 (6)	0.0503 (6)	0.0012 (4)	0.0137 (4)	−0.0026 (5)
O2	0.0298 (5)	0.0681 (8)	0.0481 (7)	0.0069 (5)	0.0153 (5)	−0.0067 (6)
O3	0.0869 (11)	0.0777 (10)	0.0787 (10)	0.0202 (8)	0.0540 (9)	0.0361 (8)
O4	0.0795 (9)	0.0357 (6)	0.0589 (8)	0.0076 (6)	0.0165 (7)	−0.0049 (6)
S1	0.0714 (3)	0.0572 (3)	0.0452 (2)	0.0234 (2)	0.0321 (2)	−0.00093 (19)

### Geometric parameters (Å, °)

**Table:**

C1—N1	1.4526 (18)	C16—H16	0.9300
C1—H1A	0.9600	C17—C22	1.406 (3)
C1—H1B	0.9600	C17—C18	1.407 (2)
C1—H1C	0.9600	C18—C19	1.406 (2)
C2—N1	1.4598 (17)	C19—C20	1.367 (2)
C2—C3	1.5050 (19)	C20—C21	1.418 (2)
C2—H2A	0.9700	C20—H20	0.9300
C2—H2B	0.9700	C21—C22	1.361 (3)
C3—C31	1.3432 (19)	C21—H21	0.9300
C3—C4	1.4830 (19)	C22—H22	0.9300
C4—O1	1.2145 (16)	C31—C32	1.454 (2)
C4—C5	1.5297 (17)	C31—H31	0.9300
C5—C6	1.5340 (19)	C32—C37	1.394 (2)
C5—C7	1.5534 (19)	C32—C33	1.396 (2)
C5—C11	1.6091 (19)	C33—C34	1.372 (2)
C6—N1	1.4512 (18)	C33—H33	0.9300
C6—H6A	0.9700	C34—C35	1.387 (2)
C6—H6B	0.9700	C34—H34	0.9300
C7—C71	1.514 (2)	C35—O4	1.363 (2)
C7—C8	1.5228 (19)	C35—C36	1.380 (2)
C7—H7	0.9800	C36—C37	1.376 (2)
C8—N2	1.4564 (18)	C36—H36	0.9300
C8—C9	1.512 (2)	C37—H37	0.9300
C8—H8	0.9800	C38—O4	1.415 (2)
C9—S1	1.8181 (19)	C38—H38A	0.9600
C9—H9A	0.9700	C38—H38B	0.9600
C9—H9B	0.9700	C38—H38C	0.9600
C10—N2	1.452 (2)	C71—C76	1.386 (2)
C10—S1	1.8245 (17)	C71—C72	1.387 (2)
C10—H10A	0.9700	C72—C73	1.387 (2)
C10—H10B	0.9700	C72—H72	0.9300
C11—N2	1.4606 (18)	C73—C74	1.375 (2)
C11—C19	1.5197 (19)	C73—H73	0.9300
C11—C12	1.5764 (19)	C74—C75	1.373 (2)
C12—O2	1.2137 (18)	C74—O3	1.374 (2)
C12—C13	1.480 (2)	C75—C76	1.383 (2)
C13—C14	1.373 (2)	C75—H75	0.9300
C13—C18	1.399 (2)	C76—H76	0.9300
C14—C15	1.405 (3)	C77—O3	1.393 (3)
C14—H14	0.9300	C77—H77A	0.9600
C15—C16	1.363 (4)	C77—H77B	0.9600
C15—H15	0.9300	C77—H77C	0.9600
C16—C17	1.411 (3)		
			
N1—C1—H1A	109.5	C18—C17—C16	115.41 (19)
N1—C1—H1B	109.5	C13—C18—C19	113.01 (14)
H1A—C1—H1B	109.5	C13—C18—C17	123.50 (16)
N1—C1—H1C	109.5	C19—C18—C17	123.45 (16)
H1A—C1—H1C	109.5	C20—C19—C18	118.70 (14)
H1B—C1—H1C	109.5	C20—C19—C11	131.68 (14)
N1—C2—C3	113.38 (11)	C18—C19—C11	109.62 (12)
N1—C2—H2A	108.9	C19—C20—C21	118.48 (16)
C3—C2—H2A	108.9	C19—C20—H20	120.8
N1—C2—H2B	108.9	C21—C20—H20	120.8
C3—C2—H2B	108.9	C22—C21—C20	122.62 (17)
H2A—C2—H2B	107.7	C22—C21—H21	118.7
C31—C3—C4	116.34 (13)	C20—C21—H21	118.7
C31—C3—C2	123.88 (13)	C21—C22—C17	120.43 (16)
C4—C3—C2	119.52 (11)	C21—C22—H22	119.8
O1—C4—C3	122.30 (12)	C17—C22—H22	119.8
O1—C4—C5	121.64 (12)	C3—C31—C32	128.77 (14)
C3—C4—C5	116.05 (11)	C3—C31—H31	115.6
C4—C5—C6	106.32 (11)	C32—C31—H31	115.6
C4—C5—C7	112.96 (11)	C37—C32—C33	117.03 (14)
C6—C5—C7	113.35 (11)	C37—C32—C31	124.23 (14)
C4—C5—C11	109.67 (10)	C33—C32—C31	118.70 (14)
C6—C5—C11	109.78 (11)	C34—C33—C32	121.66 (15)
C7—C5—C11	104.77 (10)	C34—C33—H33	119.2
N1—C6—C5	107.06 (11)	C32—C33—H33	119.2
N1—C6—H6A	110.3	C33—C34—C35	119.86 (16)
C5—C6—H6A	110.3	C33—C34—H34	120.1
N1—C6—H6B	110.3	C35—C34—H34	120.1
C5—C6—H6B	110.3	O4—C35—C36	124.61 (15)
H6A—C6—H6B	108.6	O4—C35—C34	115.64 (15)
C71—C7—C8	116.73 (11)	C36—C35—C34	119.75 (15)
C71—C7—C5	115.43 (11)	C37—C36—C35	119.76 (15)
C8—C7—C5	101.62 (11)	C37—C36—H36	120.1
C71—C7—H7	107.5	C35—C36—H36	120.1
C8—C7—H7	107.5	C36—C37—C32	121.79 (15)
C5—C7—H7	107.5	C36—C37—H37	119.1
N2—C8—C9	104.23 (12)	C32—C37—H37	119.1
N2—C8—C7	100.71 (10)	O4—C38—H38A	109.5
C9—C8—C7	119.68 (13)	O4—C38—H38B	109.5
N2—C8—H8	110.5	H38A—C38—H38B	109.5
C9—C8—H8	110.5	O4—C38—H38C	109.5
C7—C8—H8	110.5	H38A—C38—H38C	109.5
C8—C9—S1	103.98 (11)	H38B—C38—H38C	109.5
C8—C9—H9A	111.0	C76—C71—C72	116.71 (14)
S1—C9—H9A	111.0	C76—C71—C7	122.12 (13)
C8—C9—H9B	111.0	C72—C71—C7	121.16 (13)
S1—C9—H9B	111.0	C71—C72—C73	122.17 (14)
H9A—C9—H9B	109.0	C71—C72—H72	118.9
N2—C10—S1	104.21 (11)	C73—C72—H72	118.9
N2—C10—H10A	110.9	C74—C73—C72	119.70 (15)
S1—C10—H10A	110.9	C74—C73—H73	120.1
N2—C10—H10B	110.9	C72—C73—H73	120.1
S1—C10—H10B	110.9	C75—C74—O3	115.43 (16)
H10A—C10—H10B	108.9	C75—C74—C73	119.23 (16)
N2—C11—C19	112.11 (11)	O3—C74—C73	125.32 (16)
N2—C11—C12	114.09 (11)	C74—C75—C76	120.68 (16)
C19—C11—C12	101.61 (11)	C74—C75—H75	119.7
N2—C11—C5	101.37 (11)	C76—C75—H75	119.7
C19—C11—C5	116.65 (10)	C75—C76—C71	121.46 (15)
C12—C11—C5	111.58 (11)	C75—C76—H76	119.3
O2—C12—C13	127.32 (14)	C71—C76—H76	119.3
O2—C12—C11	123.90 (15)	O3—C77—H77A	109.5
C13—C12—C11	107.74 (12)	O3—C77—H77B	109.5
C14—C13—C18	119.42 (18)	H77A—C77—H77B	109.5
C14—C13—C12	132.90 (18)	O3—C77—H77C	109.5
C18—C13—C12	107.65 (13)	H77A—C77—H77C	109.5
C13—C14—C15	117.9 (2)	H77B—C77—H77C	109.5
C13—C14—H14	121.0	C6—N1—C1	113.95 (12)
C15—C14—H14	121.0	C6—N1—C2	112.90 (11)
C16—C15—C14	122.8 (2)	C1—N1—C2	110.95 (12)
C16—C15—H15	118.6	C10—N2—C8	110.49 (11)
C14—C15—H15	118.6	C10—N2—C11	120.41 (12)
C15—C16—C17	121.0 (2)	C8—N2—C11	108.76 (11)
C15—C16—H16	119.5	C74—O3—C77	118.35 (17)
C17—C16—H16	119.5	C35—O4—C38	117.49 (16)
C22—C17—C18	116.21 (16)	C9—S1—C10	93.62 (7)
C22—C17—C16	128.34 (19)		
			
N1—C2—C3—C31	152.33 (14)	C17—C18—C19—C11	177.71 (14)
N1—C2—C3—C4	−21.59 (18)	N2—C11—C19—C20	−51.1 (2)
C31—C3—C4—O1	27.6 (2)	C12—C11—C19—C20	−173.33 (15)
C2—C3—C4—O1	−158.01 (14)	C5—C11—C19—C20	65.1 (2)
C31—C3—C4—C5	−150.91 (13)	N2—C11—C19—C18	128.13 (13)
C2—C3—C4—C5	23.46 (18)	C12—C11—C19—C18	5.91 (14)
O1—C4—C5—C6	137.07 (14)	C5—C11—C19—C18	−115.64 (13)
C3—C4—C5—C6	−44.39 (15)	C18—C19—C20—C21	3.1 (2)
O1—C4—C5—C7	12.15 (18)	C11—C19—C20—C21	−177.75 (15)
C3—C4—C5—C7	−169.31 (11)	C19—C20—C21—C22	−0.9 (3)
O1—C4—C5—C11	−104.30 (15)	C20—C21—C22—C17	−1.6 (3)
C3—C4—C5—C11	74.24 (14)	C18—C17—C22—C21	1.8 (3)
C4—C5—C6—N1	66.56 (13)	C16—C17—C22—C21	−175.8 (2)
C7—C5—C6—N1	−168.75 (10)	C4—C3—C31—C32	172.66 (14)
C11—C5—C6—N1	−51.99 (13)	C2—C3—C31—C32	−1.4 (3)
C4—C5—C7—C71	87.51 (14)	C3—C31—C32—C37	34.3 (3)
C6—C5—C7—C71	−33.50 (16)	C3—C31—C32—C33	−143.31 (17)
C11—C5—C7—C71	−153.17 (11)	C37—C32—C33—C34	−4.3 (3)
C4—C5—C7—C8	−145.17 (11)	C31—C32—C33—C34	173.53 (16)
C6—C5—C7—C8	93.81 (13)	C32—C33—C34—C35	1.9 (3)
C11—C5—C7—C8	−25.85 (12)	C33—C34—C35—O4	−178.46 (17)
C71—C7—C8—N2	170.13 (12)	C33—C34—C35—C36	1.8 (3)
C5—C7—C8—N2	43.67 (12)	O4—C35—C36—C37	177.43 (17)
C71—C7—C8—C9	−76.56 (17)	C34—C35—C36—C37	−2.8 (3)
C5—C7—C8—C9	156.98 (13)	C35—C36—C37—C32	0.3 (3)
N2—C8—C9—S1	−42.06 (13)	C33—C32—C37—C36	3.2 (2)
C7—C8—C9—S1	−153.47 (11)	C31—C32—C37—C36	−174.47 (15)
C4—C5—C11—N2	120.22 (11)	C8—C7—C71—C76	−18.4 (2)
C6—C5—C11—N2	−123.32 (11)	C5—C7—C71—C76	100.91 (17)
C7—C5—C11—N2	−1.29 (12)	C8—C7—C71—C72	161.37 (14)
C4—C5—C11—C19	−1.82 (16)	C5—C7—C71—C72	−79.35 (17)
C6—C5—C11—C19	114.64 (13)	C76—C71—C72—C73	−2.3 (2)
C7—C5—C11—C19	−123.33 (12)	C7—C71—C72—C73	177.93 (15)
C4—C5—C11—C12	−117.96 (12)	C71—C72—C73—C74	1.1 (3)
C6—C5—C11—C12	−1.50 (15)	C72—C73—C74—C75	0.9 (3)
C7—C5—C11—C12	120.53 (12)	C72—C73—C74—O3	179.07 (18)
N2—C11—C12—O2	42.7 (2)	O3—C74—C75—C76	−179.83 (19)
C19—C11—C12—O2	163.57 (14)	C73—C74—C75—C76	−1.5 (3)
C5—C11—C12—O2	−71.42 (18)	C74—C75—C76—C71	0.1 (3)
N2—C11—C12—C13	−126.34 (13)	C72—C71—C76—C75	1.7 (3)
C19—C11—C12—C13	−5.49 (14)	C7—C71—C76—C75	−178.54 (18)
C5—C11—C12—C13	119.52 (12)	C5—C6—N1—C1	162.47 (12)
O2—C12—C13—C14	12.5 (3)	C5—C6—N1—C2	−69.76 (14)
C11—C12—C13—C14	−178.95 (19)	C3—C2—N1—C6	45.11 (16)
O2—C12—C13—C18	−165.29 (15)	C3—C2—N1—C1	174.43 (13)
C11—C12—C13—C18	3.29 (17)	S1—C10—N2—C8	−36.52 (14)
C18—C13—C14—C15	−1.2 (3)	S1—C10—N2—C11	−164.65 (10)
C12—C13—C14—C15	−178.8 (2)	C9—C8—N2—C10	52.90 (15)
C13—C14—C15—C16	1.1 (4)	C7—C8—N2—C10	177.50 (12)
C14—C15—C16—C17	0.1 (4)	C9—C8—N2—C11	−172.86 (11)
C15—C16—C17—C22	176.7 (2)	C7—C8—N2—C11	−48.26 (13)
C15—C16—C17—C18	−0.9 (3)	C19—C11—N2—C10	−75.52 (16)
C14—C13—C18—C19	−177.52 (16)	C12—C11—N2—C10	39.29 (18)
C12—C13—C18—C19	0.60 (18)	C5—C11—N2—C10	159.34 (12)
C14—C13—C18—C17	0.3 (3)	C19—C11—N2—C8	155.57 (11)
C12—C13—C18—C17	178.46 (15)	C12—C11—N2—C8	−89.62 (14)
C22—C17—C18—C13	−177.17 (16)	C5—C11—N2—C8	30.44 (13)
C16—C17—C18—C13	0.7 (3)	C75—C74—O3—C77	−166.0 (2)
C22—C17—C18—C19	0.5 (2)	C73—C74—O3—C77	15.8 (3)
C16—C17—C18—C19	178.39 (17)	C36—C35—O4—C38	−6.6 (3)
C13—C18—C19—C20	174.93 (14)	C34—C35—O4—C38	173.64 (19)
C17—C18—C19—C20	−2.9 (2)	C8—C9—S1—C10	19.41 (11)
C13—C18—C19—C11	−4.42 (17)	N2—C10—S1—C9	8.36 (12)

### Hydrogen-bond geometry (Å, °)

**Table:**

Cg1 and Cg2 are the centroids of the C17–C22 and C71–C76 rings, respectively.

**Table:**

*D*—H···*A*	*D*—H	H···*A*	*D*···*A*	*D*—H···*A*
C76—H76···O2^i^	0.93	2.38	3.263 (2)	159
C34—H34···O1^ii^	0.93	2.53	3.453 (2)	171
C10—H10B···O2	0.97	2.53	3.173 (2)	124
C2—H2A···Cg1^iii^	0.97	2.73	3.658 (2)	160
C38—H38B···Cg2^iv^	0.96	2.93	3.730 (2)	141

Symmetry codes: (i) −*x*, −*y*+1, −*z*; (ii) −*x*+1, *y*−1/2, −*z*+1/2; (iii) *x*, −*y*−1/2, *z*−3/2; (iv) *x*, *y*−1, *z*.

## References

[bb1] Bernstein, J., Davis, R. E., Shimoni, L. & Chang, N.-L. (1995). *Angew. Chem. Int. Ed. Engl.* **34**, 1555–1573.

[bb2] Bruker (2004). *APEX2* and *SAINT* Bruker AXS Inc., Madison, Wisconsin, USA.

[bb3] Caramella, P. & Grunanger, P. (1984). *1,3-Dipolar Cycloaddition Chemistry*, Vol. 1, edited by A. Padwa, pp. 291–312. New York: Wiley.

[bb4] James, D., Kunze, H. B. & Faulkner, D. (1991). *J. Nat. Prod.* **54**, 1137–1140. 10.1021/np50076a040 1791478

[bb5] Kobayashi, J., Tsuda, M., Agemi, K. & Vacelet, J. (1991). *Tetrahedron*, **47**, 6617–6622.

[bb6] Sheldrick, G. M. (1996). *SADABS*, University of Göttingen, Germany.

[bb7] Sheldrick, G. M. (2008). *Acta Cryst.* A**64**, 112–122. 10.1107/S0108767307043930 18156677

[bb8] Spek, A. L. (2009). *Acta Cryst.* D**65**, 148–155. 10.1107/S090744490804362X PMC2631630 19171970

